# The Role of Reticulocyte-Derived Parameters in the Detection of Iron-Restricted Erythropoiesis in the Elderly

**DOI:** 10.3390/diagnostics16060928

**Published:** 2026-03-20

**Authors:** Eloísa Urrechaga, Mónica Fernández

**Affiliations:** 1 Laboratory, Hospital Galdakao Usansolo, 48960 Galdakao, Spain; 2 Hematology, Hospital Galdakao Usansolo, 48960 Galdakao, Spain

**Keywords:** elderly, anemia, erythropoiesis, reticulocyte Hb expression, reticulocyte volume

## Abstract

**Background**: Mindray BC-6800 Plus ^TM^ (Mindray, Shenzhen, China) measures reticulocyte counts and provides the reticulocyte hemoglobin (RHe, reticulocyte Hb expression) and mean reticulocyte volume (MRV). We studied the performance of those reticulocyte-derived parameters for the detection of iron-restricted erythropoiesis in older patients, compared with standard laboratory tests. **Methods**: A total of 220 anemic patients, age > 65 years, were recruited in the context of routine health controls. Group differences were assessed using analysis of variance (ANOVA), with *p* values < 0.05 considered statistically significant. Receiver operating characteristic (ROC) curve analysis was performed to assess the diagnostic performance of RHe and MRV for detecting iron-restricted erythropoiesis. The reference standard for iron deficiency was sTfR > 52 nmol/L. A multivariable logistic regression model was constructed for iron-restricted erythropoiesis, including MRV, Ret-He and s-ferritin as independent covariates, and adjusted for inflammatory status and renal function. **Results**: Overall, 30.1% in the group had IDA and 29.0% had mixed IDA/ACD, so 59.1% had absolute or functional iron deficiency, while 40.9% had adequate iron supply. RHe and MRV values differed significantly between both groups (*p* = 0.0001). For s-ferritin, ROC analysis yielded an AUC of 0.685 (95% CI 0.606–0.767), with the best Youden index at a cut-off of 100 µg/L, corresponding to 72.5% sensitivity and 65.9% specificity. An MRV cut-off of 97.4 fL identified iron-restricted erythropoiesis with 88.2% sensitivity and 82.7% specificity (AUC 0.878, 95% CI 0.799–0.957); RHe AUC 0.860, 95% CI 0.777–0.947; cut-off 30.4 pg; sensitivity 82.4%, specificity 79.8%). In multivariable logistic regression adjusted for CRP and eGFR, s-ferritin was not an independent predictor of iron-restricted erythropoiesis, whereas MRV and RHe remained significant. The overall model demonstrated good discrimination, with an AUC 0.808 (95% CI 0.804–0.814). **Conclusions**: RHe and MRV are reliable parameters for assessing iron supply to erythropoiesis in older patients and can assist in distinguishing iron-restricted erythropoiesis in complex, inflammation-driven settings.

## 1. Introduction

Anemia is the most common hematological disorder encountered in geriatric practice. Its prevalence, in the general ambulatory population, has been evaluated at between 10 and 15% after 65 years of age and more than 20% beyond 85 years of age. Anemia is associated with increased morbidity in terms of cardiac complications, cognitive decline, frailty, hospitalizations, impaired quality of life and increased mortality [1].

Guidelines for anemia in older adults recommend structured evaluation rather than accepting anemia as “physiologic.” First-line work-up typically includes complete blood count (CBC), s-ferritin, B12, folate, renal function, and C-reactive protein, due to the multifactorial nature of anemia in this age group [2,3].

Approximately one third of cases of anemia in the older age group are attributable to iron deficiency (ID), which is common in chronic diseases such as heart failure (HF), chronic kidney disease (CKD) or cancer. Independently of anemia, ID is also associated with more rapid clinical worsening in HF, non-dialysis CKD and cancer, and is a risk factor of mortality in patients with heart failure and CKD [4].

ID is a broad definition encompassing decreased total body iron (absolute deficiency) as well as reduced iron supply for erythropoiesis and/or other organs (functional iron deficiency, FID). The former is related with iron deficiency anemia (IDA), the latter with anemia of chronic disease (ACD), when inflammation impairs the iron release from storage sites, limiting the iron available for erythropoiesis [5].

Comprehensive iron status and erythropoiesis evaluation requires a combination of tests and clinical indicators. The most useful biomarkers for assessing iron status are s-ferritin, which reflects iron stores, and transferrin saturation (TSAT), which is indicative of the iron transported in the circulation and available for cell metabolism. But certain limitations affect those traditional biochemical markers, leading to diagnostic uncertainty, particularly in chronic inflammatory and complex clinical settings, frequent in older patients, where the values do not accurately reflect iron status and/or availability [6].

The frailty of the elderly, who often suffer diverse comorbidities, indicates the importance of better evaluating ID and erythropoiesis requirements in this population. However, there is not a consensus definition of ID in the elderly and the use of specific thresholds for biomarkers of iron metabolism is still a controversial matter [7].

The reticulocyte count is crucial for the investigation of the erythropoiesis status, identifying an adequate bone marrow response. Contemporary counters can also provide additional derived parameters [8]. Reticulocyte hemoglobin content is recognized as a reliable marker for the detection of iron-restricted erythropoiesis. It is not affected by acute-phase response, so it is an early indicator of functional iron deficiency (FID), and can aid to prevent progression to overt anemia and in monitoring the effectiveness of treatments [9,10,11].

The Mindray BC-6800 Plus ^TM^ automated hematology analyzer employs advanced reticulocyte counting technology applying flow cytometry principles. This system not only measures reticulocyte counts but also provides the reticulocyte hemoglobin (RHe, reticulocyte Hb expression) [12]. With the new software version v 2.2.0, it can report mean reticulocyte volume (MRV). The reference range of MRV in healthy subjects, anemia and correlation with the RHe values have been recently reported [13].

In elderly patients, definitions of iron deficiency and iron-restricted erythropoiesis remain heterogeneous, and conventional markers such as ferritin and transferrin saturation are frequently confounded by chronic inflammation and multimorbidity.

Under iron-restricted erythropoiesis, hemoglobin synthesis in late erythroblasts is impaired, which leads to the release of smaller and hypochromic reticulocytes into the circulation. As a consequence, MRV decreases in parallel with reticulocyte hemoglobin content, so that MRV reflects the interplay between iron availability and erythroid maturation in the bone marrow.

Data on Mindray-derived reticulocyte parameters in this age group are particularly scarce, despite their potential to provide an early and functionally oriented assessment of erythroid iron supply. Therefore, our study aimed to establish optimal cut-off values for mean reticulocyte volume (MRV) and reticulocyte hemoglobin content (RHe) for detecting iron-restricted erythropoiesis in older outpatients, using soluble transferrin receptor (sTfR) as the reference standard, and to compare their diagnostic performance with that of conventional iron markers in this population.

## 2. Materials and Methods

This study represents a cross-sectional diagnostic accuracy analysis performed on prospectively collected samples from elderly outpatients undergoing routine laboratory testing.

### 2.1. Patients

The study was conducted as a prospectively enrolled cohort of elderly outpatients, with cross-sectional analysis of baseline hematologic and biochemical parameters.

Inclusion criteria were: anemia (Hb < 120 g/L in women, Hb < 130 g/L in men) of all types, outpatients age > 65 years. Those patients receiving iron supplementation or red cell transfusions were excluded from the study. Those with leukemia, myeloma, myelodysplastic syndromes, folate or cobalamin deficiency, and hemolytic anemia or hemoglobinopathies were also excluded.

After the requested tests had been completed, we used the residual samples for the present study. This practice is in accordance with the guidelines established by the Ethic Committee at Galdakao-Usansolo Hospital, so the study obtained the ethics approval from the Research Ethics Committee in Barrualde-Interior District IHO (Biscay, Spain), No. 1024/24.

The analyses were requested as part of the routine health controls; neither doses nor analysis requested nor visits to the clinicians were changed. The diagnostics, clinical data, comorbidities, treatments and doses of each patient were retrieved from their laboratory and medical records.

### 2.2. Analytical Methods

The samples were obtained in the course of routine analysis and collected in K2 EDTA anticoagulant tubes (Vacutainer^TM^ Becton-Dickinson, Rutherford, NJ, USA), and were run in the Mindray BC-6800 Plus analyzer within 4 h of collection. C-reactive protein (CRP), s-iron, transferrin, s-ferritin and soluble transferrin receptor (sTfR) were assayed in a Cobas c 8000 (Roche Diagnostics, Basel, Switzerland) analyzer.

sTfR ≥ 52 nmol/L was used as the reference standard for the detection of iron-restricted erythropoiesis, while s-ferritin, transferrin saturation (TSAT) and CRP were used to classify patients into IDA, ACD and mixed ACD/IDA, based on the following criteria [5]:

Iron deficiency anemia (IDA): when they presented low Hb (male < 130 g/L and female < 120 g/L), transferrin saturation (TSAT) < 20%, serum ferritin (s-ferritin) < 30 µg/L and no signs of inflammation C-reactive protein (CRP) < 5.0 mg/L.

Anemia of chronic disease (ACD): these patients had evidence of chronic inflammation: Hb < 130 g/L for male and <120 g/L for female, TSAT < 20%, s-ferritin > 100 µg/L, sTfR < 52 nmol/L.

ACD with concomitant IDA (ACD/IDA): Hb < 130 g/L for male and <120 g/L for female, TSAT < 20%, s-ferritin 30–100 µg/L and chronic inflammation, sTfR > 52 nmol/L.

### 2.3. Statistical Analysis

Normality of continuous variables was assessed using the Kolmogorov–Smirnov test. Differences among groups were assessed with analysis of variance (ANOVA). Post hoc testing using Scheffe’s test was applied for those parameters that had obtained a significant difference, to provide specific information on which values are significantly different from each other; *p* < 0.05 was considered to be statistically significant.

Receiver operating characteristic (ROC) curve analysis was utilized to verify the diagnostic performance of RHe and MRV for detecting iron-restricted erythropoiesis. The gold standard for iron deficiency was sTfR > 52 nmol/L.

Pairwise comparisons of ROC curves were performed using DeLong’s test.

A multivariable logistic regression model was constructed for iron-restricted erythropoiesis, including MRV, Ret-He and s-ferritin as independent covariates, and adjusted for inflammatory status (CRP < 5.0 vs. ≥ 5.0 mg/L) and renal function (eGFR < 60 vs. ≥ 60 mL/min/1.73 m^2^).

The results are presented as odds ratios and the model performance was evaluated using the area under the ROC curve and standard goodness-of-fit diagnostics.

Statistical software package SPSS version 29.0 for windows was used for statistical analysis (SPSS; Chicago, IL 60606, USA).

## 3. Results

Table 1 summarizes the clinical characteristics of the group (*n* = 220). In total, 18.7% of our patients suffered kidney disease, and 22.3% had abnormal renal function (eGFR < 60 mL/min/1.73 m^2^).

**Figure 1:**
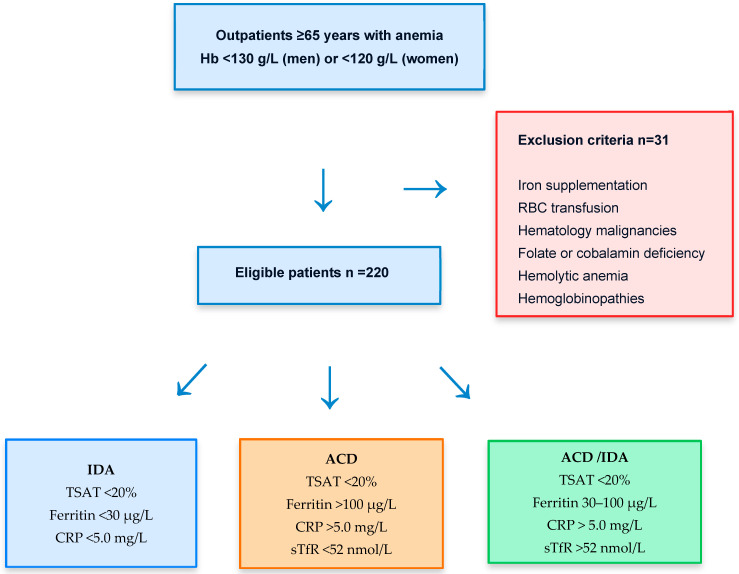
Patient selection flow chart summarizes the study workflow, including application of inclusion and exclusion criteria, and subsequent diagnostic classification. After exclusions, patients were grouped into iron deficiency anemia (IDA), anemia of chronic disease (ACD), and mixed IDA/ACD based on ferritin, transferrin saturation (TSAT), C-reactive protein (CRP), and soluble transferrin receptor (sTfR), as detailed in the Section 2.

A flowchart (Figure 1) summarizes the study workflow, including initial patient screening, application of inclusion and exclusion criteria, and subsequent diagnostic classification. Thirty-one patients were excluded from the study.

Table 2 presents the laboratory parameters for the assessment of iron status in the group. Patients were diagnosed with IDA, ACD and concomitant ACD/IDA, based on the previously mentioned criteria [5].

**Figure 2:**
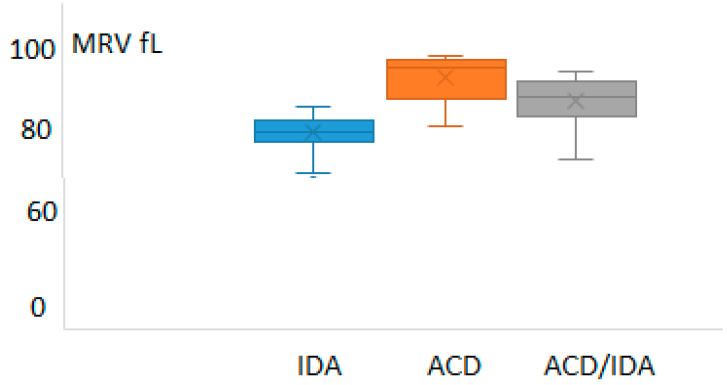
Values of mean reticulocyte volume (MRV) in the patients suffering from iron deficiency anemia (IDA), anemia of chronic disease (ACD) and anemia of chronic disease with concomitant IDA (ACD/IDA).

**Figure 3:**
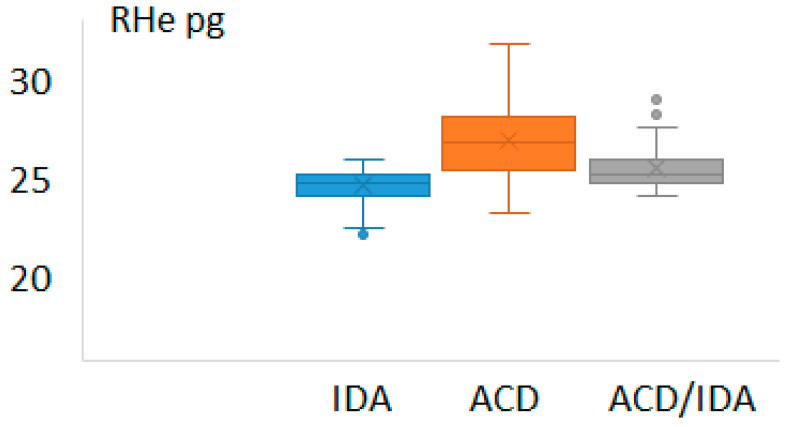
Values of reticulocyte hemoglobin (RHe) in the patients suffering from iron deficiency anemia (IDA), anemia of chronic disease (ACD) and anemia of chronic disease with concomitant IDA (ACD/IDA).

The values of the reticulocyte-derived indices (MRV and RHe) in those groups are depicted in Figure 2 and Figure 3, respectively.

The results in the IDA group reflected a state of iron depletion: low ferritin, low iron availability (low TSAT) and iron-restricted erythropoiesis (low RHe and MRV). Patients with ACD or ACD/IDA showed the typical pattern with normal or high s-ferritin. Based on sTfR values, the ACD group was divided into those with adequate iron supply and those with functional iron deficiency.

In total, 30.1% suffered with IDA and 29.0% mixed IDA and ACD; thus, 59.1% suffered from iron deficiency, absolute or functional, while 40.9% had an adequate supply, as higher TSAT and lower sTfR values show. Both groups had RHe and MRV values significantly different *p* = 0.0001.

The median s-ferritin in the IDA group was significantly lower compared to patients with inflammation, *p* < 0.001.

Regarding ROC analyses, “iron-restricted erythropoiesis” was defined by sTfR ≥ 52 nmol/L, irrespective of the underlying clinical category (IDA, ACD or mixed IDA/ACD). Thus, patients with absolute or functional iron deficiency (IDA + mixed IDA/ACD) were grouped as having iron-restricted erythropoiesis, while those with sTfR < 52 nmol/L were considered to have a sufficient iron supply.

The ROC analysis result for s-Ferritin was area under curve (AUC) 0.685 (95% confidence interval, CI: 0.606–0.767). The sensitivity and specificity at different cut-offs are presented in Table 3. The best Youden index was obtained at a cut-off level 100 µg/L, sensitivity 72.5% and a specificity of 65.9%.

At a cut-off MRV 97.4 fL, iron-restricted erythropoiesis can be diagnosed with sensitivity 88.2% and specificity 82.7%. The area under the curve was 0.878 (95% CI 0.799–0.957). These values are in agreement with the RHe results, AUC 0.860 (95% CI 0.777–0.947) *p* = 0.09. At a cut-off RHe 30.4 pg, iron-restricted erythropoiesis could be diagnosed with sensitivity 82.4% and specificity 79.8%. Pairwise comparisons of ROC curves confirmed that the AUCs for MRV and RHe differed significantly from that of s-ferritin (*p* < 0.001), indicating that reticulocyte-derived indices significantly outperform s-ferritin for detecting iron-restricted erythropoiesis.

We performed an exploratory stratified ROC analysis according to renal function (eGFR ≥ 60 vs. <60 mL/min/1.73 m^2^). The AUCs for MRV and RHe remained high and of similar magnitude across strata, whereas ferritin performance deteriorated in the high-CRP and reduced-eGFR subgroups.

**Figure 4:**
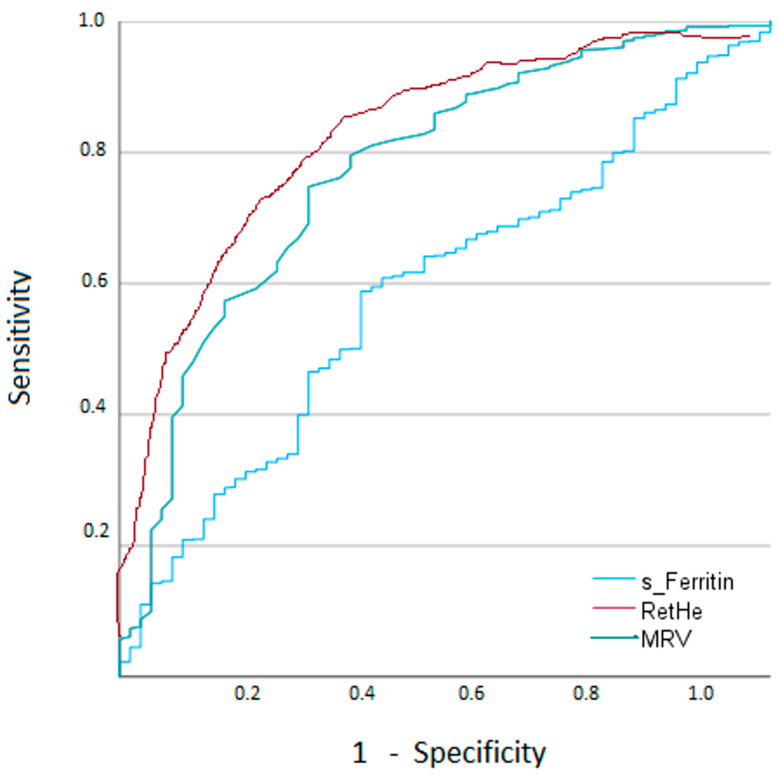
ROC analyses of s-Ferritin, MRV and RHe for the detection of iron-restricted erythropoiesis, defined by sTfR ≥ 52 nmol/L, irrespective of the underlying clinical category (IDA, ACD or mixed IDA/ACD).

Figure 4 summarizes ROC curves for s-Ferritin, MRV and RHe.

In multivariable logistic regression adjusted for CRP and eGFR, s-ferritin was not an independent predictor of iron-restricted erythropoiesis, whereas MRV and RHe remained significant. MRV had an odds ratio of 0.806 (95% CI 0.801–0.811) per fL decrease (*p* < 0.001) and RHe had an odds ratio of 0.849 (95% CI 0.844–0.853) per pg decrease (*p* < 0.001). The overall model demonstrated good discrimination, with an AUC 0.808 (95% CI 0.804–0.814).

## 4. Discussion

The population in Europe is aging, with the average age steadily increasing. In Spain, the percentage of people over 65 years old stands at around 20.4% in 2025 [14]. Our Health Organization serves a geographically dispersed population covering areas of Bizkaia and the Ayala Valley in Alava in the North Spain. According to official data in 2025, out of a total population of 337,680 inhabitants, 21.5% of them are between 65 and 92 years or older, reflecting a significant proportion of elderly people [15].

In older patients, iron deficiency is often multifactorial (occult blood loss, medications, malnutrition, inflammation). Detection therefore usually starts either from anemia on CBC or from symptoms such as fatigue, reduced exercise tolerance, falls, or frailty in high-risk patients [16].

In our group, the prevalence of comorbidities was high: in total, 70% of the patients suffered from inflammation, 30.1% suffered from IDA and 29.0% mixed IDA and ACD; thus, 59.1% suffered from either absolute or functional iron deficiency.

The detection of iron deficiency in the elderly relies on integrating history, CBC morphology, and iron studies, but inflammation and multimorbidity make interpretation trickier than in younger patients [6,17].

The diagnosis of iron deficiency is important because the proper iron therapy can improve the symptoms as well as possibly indicate the occult gastrointestinal pathology, such as malignancy. Although the confirmation of the absence of iron storage in the bone marrow aspirate sample should be the “gold standard” for the diagnosis of iron deficiency, the value of less invasive tests assessing iron storage in general populations has been well established. The serum ferritin assay is the best single blood test for the diagnosis of iron deficiency [17].

The interpretation in older adults is complicated sometimes because s-ferritin tends to increase with age and then plateau after about 60 years, even without overt disease, partly due to chronic low-grade inflammatory activity. In geriatric cohorts free of overt infection or malignancy, ferritin correlates more strongly with inflammatory markers than with iron status, meaning normal or moderately elevated ferritin can mask depleted iron stores [18].

This implies that the application of the cut-off point derived from younger populations to the elderly is inappropriate. In older adults, many guidelines and geriatric sources suggest investigating for iron deficiency when ferritin is below about 50 µg/L, and using a broader “suspicious” zone up to 100 µg/L, because comorbidity and inflammation can mask deficiency by elevating ferritin [17,19]. Conversely, in hospital or high-inflammation settings, raising the “rule-out” ferritin to 100 µg/L improves sensitivity for iron deficiency, but with a marked loss of specificity, illustrating how inflammation pushes the useful diagnostic window upward [20,21,22].

In the present study, the diagnostic performance of s-ferritin in the diagnosis of iron deficiency was found to be poor, with the best Youden index obtained at a cut-off 100 µg/L.

In this elderly multimorbid cohort with frequent low-grade inflammation, ROC analysis identified a ferritin cut-off of 100 µg/L as providing the best balance between sensitivity and specificity for detecting iron-restricted erythropoiesis. This data-driven threshold differs from the conventional 30 µg/L ferritin level typically used to define absolute iron deficiency in younger, non-inflamed populations. We explicitly note that, in older patients with chronic inflammatory conditions, higher ferritin thresholds (around 50–100 µg/L) are often recommended to improve sensitivity for iron deficiency, which is consistent with our finding that 100 µg/L was optimal in this setting.

Hence, our study suggests that, for our population, the conventional threshold value for the diagnosis of iron deficiency in young adults could not be appropriate for the accurate evaluation of the elderly population.

Using higher ferritin and TSAT thresholds, and second-line markers such as soluble transferrin receptor or an sTfR–ferritin index, improves sensitivity in this age group [17]. Incorporating sTfR (or an sTfR–ferritin index) as a reflex test in elderly anemias with inconclusive ferritin/TSAT, and using higher ferritin thresholds (<100 µg/L) for defining iron deficiency in multimorbid patients, would improve detection without substantially increasing invasive procedures [23].

Regarding CBC and red cell indices, microcytosis and hypochromia are traditional clues but are often absent in older adults, especially with concurrent inflammation or mixed etiologies. Many elderly patients with bone marrow-proven iron deficiency have normocytic or even mildly macrocytic indices, so a normal MCV cannot exclude iron deficiency [1].

Integrating reticulocyte hemoglobin with ferritin, transferrin saturation, and inflammatory markers has been proposed to refine the classification of anemia in the elderly, especially to separate absolute/functional iron deficiency from ACD and other causes [24,25].

Different analyzers report homologous parameters (CHr on some flow cytometry-based platforms, Ret-He on Sysmex, MCHr or similar on others), all intended as functional markers of iron supply to erythropoiesis, serving as an early marker for iron availability, which is particularly useful in elderly patients, where anemia is common due to multifactorial causes and can guide timely iron supplementation [26,27].

In previous reports, Karlsson studied anemic elderly patients (>60 years) with bone marrow iron as the gold standard; CHr at 30.5 pg had sensitivity 93% and specificity 69% for iron deficiency anemia, and CBCs were run with ADVIA counters (Siemens, Erlangen, Germany) [26]. Despite the use of different technology, our results are in agreement with those data on reticulocyte Hb; MVR cut-offs for IRE detection has not been previously reported for this population.

Joosten et al., using Sysmex counters, found that a Ret-He cut-off value of 26.0 pg had a sensitivity 85% and specificity 69% based on its optimal combination [27]. The optimal cut-off was lower in this study, which may be due to the inclusion criteria; in this study, patients were admitted to the acute geriatric ward.

Nevertheless, it must be highlighted that, despite its proven usefulness for the early diagnosis of iron deficiency and the monitoring of erythropoiesis status [10,28], several factors drive the urgent need for better alignment.

The standardization and harmonization of reticulocyte hemoglobin are critical because significant analytical differences between diagnostic manufacturers currently limit universal application in daily practice, and it is necessary to ensure that clinical outcomes are consistent across different laboratories and diagnostic platforms [29].

The International Council for Standardization in Hematology (ICSH) and the International Society for Laboratory Hematology (ISLH) have established working groups to address these discrepancies. Efforts are focused on method comparison and statistical recalibration, while the challenges are the technological variability and lack of unified reference materials, which present differences among those instruments [30]. Therefore, reference ranges should be determined according to the technologies or brands, and these could not be interchangeable in clinical practice.

The study has some weaknesses. First, this is a single-center study, so multicentric evaluations should be conducted to confirm the optimal cut-offs. Second, our cohort consisted exclusively of elderly outpatients attending routine clinical controls, with relatively stable clinical status and limited acute morbidity. As a result, the generalizability of our findings to acutely ill inpatients or to institutionalized, highly frail geriatric populations is restricted, and the proposed MRV and RHe cut-off values should be extrapolated to these settings with caution. Additional studies in hospitalized and higher-dependency elderly patients are warranted to confirm the applicability and clinical usefulness of these cut-offs across the full spectrum of geriatric care.

Inflammation and renal function are key modifiers in this elderly cohort, so a stratified multivariable logistic regression model was applied and the reliability of MRV and RHe as robust predictors of iron-restricted erythropoiesis was verified, AUC 0.808 (95% CI 0.804–0.814).

Although we used sTfR ≥ 52 nmol/L as the reference standard, this marker is not a universally accepted gold standard for iron-restricted erythropoiesis. Bone marrow iron staining remains the classical reference, but it is invasive and not feasible in routine outpatient practice, particularly in elderly patients. Moreover, sTfR levels can be influenced and overall erythropoietic activity, which should be considered when interpreting our results, can be considered a limitation of the study.

On the other hand, this is one of the few studies focusing specifically on the performance and cut-off values of reticulocyte indices in an elderly cohort, taking into account comorbidities, polypharmacy, and the frequent coexistence of chronic inflammation.

MRV reflects the volume of circulating reticulocytes and thus the efficiency of hemoglobinization in the late stages of erythropoiesis. In iron-restricted states, impaired iron delivery to erythroid precursors limits hemoglobin synthesis, leading to the production of smaller, hypochromic reticulocytes and a consequent decrease in MRV. This early morphologic change precedes overt microcytosis in mature red cells, which explains why MRV can detect iron-restricted erythropoiesis before conventional red cell indices become abnormal. The close pathophysiological link between iron supply, reticulocyte size, and subsequent red cell microcytosis supports the strong diagnostic performance of MRV observed in our study and its potential role as an early marker of functional iron deficiency.

Nevertheless, analytical issues such as the analyzer brand should be considered in order to understand the transferability of the proposed cut-offs.

MRV is a derived parameter whose absolute values may be influenced by platform-specific analytical factors, including differences in optical or impedance technology, gating strategies for reticulocyte identification, and proprietary algorithms for volume calculation. As a result, the MRV cut-off identified in our cohort should be considered analyzer-specific and may not be directly transferable to other instruments without appropriate validation. Nonetheless, the underlying concept of reduced reticulocyte volume in iron-restricted erythropoiesis is platform-independent, and MRV remains an attractive reticulocyte-derived parameter because it integrates early morphological changes with routine hematology testing. Future studies should evaluate harmonization strategies and establish platform-adapted cut-offs to facilitate broader implementation.

Our study investigates advanced reticulocyte parameters as accessible, automated markers to improve early detection of iron deficiency, in the complex clinical context of older patients. The multivariable logistic regression model demonstrated the independent contribution of reticulocyte-derived parameters for the identification of iron-restricted erythropoiesis in the elderly outpatient population by showing that both MRV and RHe remain significant predictors of iron-restricted erythropoiesis after adjustment for inflammation, ferritin and renal function.

These indices are directly generated by modern hematology analyzers and can be integrated into existing workflows without additional burden on laboratory resources.

## 5. Conclusions

The treatment of iron deficiency in older subjects improves their quality of life. Among the types of anemia in the elderly, a peculiar role seems to be played by the anemia associated with chronic inflammation, which remains the most complex form of anemia to treat.

Anemia and iron-restricted erythropoiesis are highly prevalent in geriatric populations and are associated with functional decline, increased morbidity, and mortality, yet they remain underdiagnosed and often misclassified in routine practice, because the evaluation of erythropoiesis status is particularly challenging because most of the biochemical markers for iron metabolism are affected by the acute-phase reaction.

Our findings suggest that incorporating these parameters into routine complete blood count interpretation may enhance diagnostic accuracy, refine etiologic classification, and support more timely and targeted therapeutic decisions in geriatric anemia management. We believe these results will be of particular interest to clinicians and laboratory professionals working in hematology, internal medicine, and geriatric care.

## Figures and Tables

**Table 1:** Clinical characteristics of the group (*n* = 220).

Patients’ Characteristics	Percentage
Age years, mean (range)	80.5 (65–86)
Women %	64.4
Arterial hypertension	43.2
Diabetes	27.5
Heart Failure	21.8
Ischemic heart disease	21.3
Chronic kidney disease	18.7
Cancer	10.5
Anticoagulant therapy	41.1

**Table 2:** Laboratory parameters for the assessment of erythropoiesis and iron status in the study group, reported as mean (standard deviation).

Variable	IDA *n* = 66	ACD/IDA Mixed *n* = 64	ACD *n* = 90	*p* IDA/Mixed	*p* Mixed/ACD	*p* IDA/ACD
RBC (10^12^/L)	4.2 (0.53)	3.7 (0.8)	3.9 (0.85)	0.0001	0.0048	0.0001
Hb (g/L)	99 (11)	107 (15)	109 (17)	0.0001	0.1791	0.0001
MCV (fL)	75.3 (3.5)	87.2 (9.9)	90.3 (6.4)	0.0001	0.2851	0.0001
MCH (pg)	21.2 (1.7)	25.2 (3.3)	29.1 (2.1)	0.0001	0.0001	0.0001
MCHC (g/L)	317 (10)	319 (13)	333 (15)	0.3696	0.0001	0.0001
Ferr (µg/L)	15 (22)	262(128)	235 (155)	0.0001	0.0076	0.0001
Iron (µmol/L)	5.4 (3.6)	9.9 (7)	10.6 (9)	0.0001	0.0032	0.0001
Transf (g/L)	2.78 (0.27)	1.89 (0.58)	2.09 (0.59)	0.0001	0.0001	0.0001
TSAT (%)	9 (5)	21 (12)	19 (11)	0.0001	0.080	0.0001
sTfR (nmol/L)	55.5 (13)	49.1 (12)	19.9 (18)	0.0180	0.0001	0.0001
Reticulocyte (%)	1.8 (1.1)	2.1 (1.8)	2.0 (1.3)	0.0278	0.2164	0.1292
RHe (pg)	24.5 (3.5)	26.3 (4.5)	29.6 (4.4)	0.0001	0.0027	0.0001
MRV (fL)	79.5 (14.2)	88.9 (15.1)	96.5 (18.3)	0.0001	0.0017	0.0001
CRP (mg/L)	1.5 (0.85)	8.8 (3.8)	9.8 (4.6)	0.0001	0.061	0.0001

RBC, red blood cell; Hb, hemoglobin; MCV, mean cell volume; MCH, mean cell hemoglobin; MCHC, mean cell hemoglobin concentration; Ferr, s-ferritin; Transf, transferrin; TSAT, transferrin saturation; sTfR, soluble transferrin receptor; RHe, reticulocyte Hb expression; MRV, reticulocyte volume; CRP, C-reactive protein.

**Table 3:** ROC analysis for the detection of iron-restricted erythropoiesis s-ferritin rendered area under curve 0.685 (95% confidence interval: 0.606–0.767). Sensitivity and specificity at diverse cut-offs.

s-Ferritin µg/L	Sensitivity %	Specificity %
15	18.8	95.5
30	28.1	92.2
50	61.9	70.3
100	72.5	65.9

## Data Availability

The original contributions presented in this study are included in the article. Further inquiries can be directed to the corresponding author.
